# Distribution of a Knockdown Resistance Mutation (L1014S) in *Anopheles gambiae* s.s. and *Anopheles arabiensis* in Western and Southern Kenya

**DOI:** 10.1371/journal.pone.0024323

**Published:** 2011-09-09

**Authors:** Hitoshi Kawada, Kyoko Futami, Osamu Komagata, Shinji Kasai, Takashi Tomita, George Sonye, Cassian Mwatele, Sammy M. Njenga, Charles Mwandawiro, Noboru Minakawa, Masahiro Takagi

**Affiliations:** 1 Department of Vector Ecology and Environment, Institute of Tropical Medicine, Nagasaki University, Nagasaki, Japan; 2 The Global Center of Excellence Program, Nagasaki University, Nagasaki, Japan; 3 National Institute of Infectious Diseases, Tokyo, Japan; 4 Springs of Hope, Mbita, Kenya; 5 Eastern and Southern Africa Center of International Parasite Control, Nairobi, Kenya; 6 Kenya Medical Research Institute, Nairobi, Kenya; Institut national de la santé et de la recherche médicale - Institut Cochin, France

## Abstract

In Kenya, insecticide-treated mosquito nets (ITNs) distributed to pregnant women and children under 5 years old through various programs have resulted in a significant reduction in malaria deaths. All of the World Health Organization-recommended insecticides for mosquito nets are pyrethroids, and vector mosquito resistance to these insecticides is one of the major obstacles to an effective malaria control program. *Anopheles gambiae* s.s. and *Anopheles arabiensis* are major malaria vectors that are widely distributed in Kenya. Two point mutations in the voltage-gated sodium channel (L1014F and L1014S) are associated with knockdown resistance (*kdr*) to DDT and pyrethroids in *An. gambiae* s.s. While the same point mutations have been reported to be rare in *An. arabiensis*, some evidence of metabolic resistance has been reported in this species. In order to determine the distribution of the point mutation L1014S in *An. gambiae* s.s. and *An. arabiensis* in southern and western Kenya, we collected larvae and screened for the mutation by DNA sequencing. We found high allelic and homozygous frequencies of the L1014S mutation in *An. gambiae* s.s. The L1014S mutation was also widely distributed in *An. arabiensis*, although the allelic frequency was lower than in *An. gambiae* s.s. The same intron sequence (length: 57 base) found in both species indicated that the mutation was introgressed by hybridization. The allelic frequency of L1014S was higher in both species in western regions, demonstrating the strong selection pressure imposed by long-lasting insecticide-treated nets (LLITN)/ITN on the *An. gambiae* s.s. and *An. arabiensis* populations in those areas. The present contribution of the L1014S mutation to pyrethroid resistance in *An. arabiensis* may be negligible. However, the homozygous frequency could increase with continuing selection pressure due to expanded LLITN coverage in the future.

## Introduction

Insecticide-treated nets (ITNs) have become a major tool in the Roll Back Malaria movement advocated by the World Health Organization in 1998 [1]. In Kenya, ITNs have mainly been distributed to pregnant women and children under 5 years old by the Kenya Ministry of Health and non-governmental organizations [2], [3]. Consequently, ITN coverage for children under 5 years old has increased rapidly from 7% in 2004 to 67% in 2006; this increase has been associated with a 44% reduction in malaria deaths [4].

Pyrethroid is the general term for a group of synthetic chemicals that are structurally modified from natural pyrethrins derived from Chrysanthemum flowers. Pyrethroid insecticides are widely used due to their high safety for mammals and efficacy for killing insects; they make up 40% of the insecticides used globally each year for indoor residual spraying against malaria vectors, and 100% of the World Health Organization-recommended insecticides for the treatment of mosquito nets are pyrethroids [5]. Pyrethroids are expected to remain the predominant vector-controlling agent for decades to come, since at present there are no suitable chemical substitutes. Therefore, pyrethroid resistance is a serious problem for vector control programs.

*Anopheles gambiae* Giles s.l. is one of the major malaria vectors in Africa, where more than 90% of the world current annual malaria incidence occurs. *Anopheles gambiae* s.s. and *Anopheles arabiensis* are widely distributed and are major vectors in Kenya. Two point mutations in the voltage-gated sodium channel have been shown to be associated with knockdown resistance (*kdr*) to DDT and pyrethroids in *An. gambiae* s.s. One mutation involves a leucine (TTA)-to-phenylalanine (TTT) substitution at residue 1014 of the gene (L1014F), and the other mutation involves a leucine (TTA)-to-serine (TCA) substitution at the same residue (L1014S) [6]. The L1014F mutation is the only allele present west of 10° W longitude in Africa, while L1014S is found in regions both west and east of 10° W, including Kenya [6]. While the coexistence of L1014F and L1014S has been reported in several countries in central Africa, L1014F has not been reported to date in *An. gambiae* s.s. in Kenya.

Recently, several authors have reported a causal relationship between the high ITN coverage due to mass campaigns and an increase in the *kdr* frequency in *An. gambiae* s.s. [7], [8], [9]. However, the point mutations described above have been reported to be rare in *An. arabiensis* [7], [10], [11], [12], [13], [14], [15] with the exception of an Ethiopian case [16], [17]. By contrast, metabolic resistance caused by the enhancement of P450 or glutathione S-transferase activity has been reported in this species [11], [15].

The objective of the present study was to investigate the distribution of *kdr* mutation alleles in *An. gambiae* s.s. and *An. arabiensis* in southern and western Kenya and to elucidate the relationships between their distributions and the other social, parasitological, and operational parameters, such as ITN coverage, malaria incidence rate, and other factors.

## Materials and Methods

### Collection of mosquito larvae

Anopheline mosquito larvae were periodically collected from the potential breeding habitats along the national road in the southern and western parts of Kenya (N 0° 57.46′–S 3° 41.963′, E 36° 43.698′–E 40° 0.261′) during the date ranges May 12, 2008 to December 11, 2010. The collection time was 15–30 min when Anopheline larvae could be found by preliminary 10 times dipping of the water in the habitats. In small and shallow habitats, larvae were directly collected with pipettes. The geographical positions of the collection points were recorded with a global positioning system as well as some habitat characteristics (habitat size, depth, canopy coverage, vegetation coverage, algae abundance, and deposit amount).

Collected fourth instar larvae were placed in 1.5-ml plastic vials containing 100% ethanol and stored at −20°C until analysis. When larvae were collected in earlier instar stages, they were reared in the laboratory until they reached the fourth instar.

### Extraction of DNA

The DNA of morphologically identified larvae was extracted using an ethanol precipitation method. Each mosquito larva was homogenized with a microtube pestle in a 1.5-ml tube containing 100 µl grinding buffer (0.8 M NaCl, 0.16 M sucrose, 0.13 M EDTA, 0.124 M Trizma base, and 0.5% SDS). The homogenate was incubated at 65°C for 30 min. The incubated sample was mixed with 14 µl of 8 M potassium acetate and kept on ice for 30 min. The cooled sample was centrifuged at 15,000 rpm for 10 min to remove debris. The supernatant was transferred to new tubes, mixed with 200 µl of cold 95% ethanol, and stored at −20°C for over 2 h. The cooled sample was centrifuged at 15,000 rpm for 20 min, and the supernatant was discarded. The precipitate was rinsed first with 70% ethanol and then with 95% ethanol. The rinsed precipitate was resuspended in 100 µl of sterile water.

### Species identification

Collected larvae were examined microscopically to distinguish the *An. gambiae* species complex from other Anopheline species based on the identification keys of Gillies and Coetzee [18]. The extracted DNA was identified using the multiplex polymerase chain reaction (PCR) method described by Scott et al. [19].

### Detection of point mutations in the voltage-gated sodium channel

PCR and direct DNA sequencing were used to identify point mutations at L1014. The extracted DNA for the species identification was used for the detection. Initial fragment amplification was carried out using primers AGKF1 (5′-CATGATCTGCCAAGATGGAA-3′) and AGKR1 (5′-GTTGGTGCAGACAAGGATGA-3′). The PCR reaction contained 4 µl of REDExtract-N-AmpTM Ready Mix (SIGMA), 0.5 µM of each primer, and 1 µl of the DNA template in a total volume of 10 µl. PCR was performed under the following conditions: 94°C for 3 min, followed by 35 cycles of 94°C for 15 s, 55°C for 30 s, and 72°C for 30 s, followed by 72°C for 10 min. Amplified fragments of the expected size were purified using ExoSAP-IT (USB Corporation, Cleveland, OH, USA) at 37°C for 30 min and then 80°C for 15 min. DNA sequencing was performed using primer Dg1 (5′-TGGATHGARWSHATGTGGGAYTG-3′). A BigDye Terminator v. 3.1 Cycle Sequencing Kit (Applied Biosystems Japan, Ltd., Tokyo, Japan) was used for DNA sequencing according to the manufacturer's instructions. Two µmol of the primer was added to a tube in a total reaction volume of 10 µl. PCR was performed under the following conditions: 96°C for 1 min followed by 25 cycles of 96°C for 10 s, 50°C for 5 s, and 60°C for 2 min. Ethanol precipitation was performed by adding 1 µl each of 0.125-M EDTA solution and 3 M sodium acetate solution and 25 µl of 100% ethanol to a 5-µl sample of the above reaction. The sample was centrifuged at 4°C at 3,000×*g* for 30 min, after which the supernatant was removed and 35 µl of 70% ethanol was added. The resulting mixture was centrifuged at 4°C at 3,000×*g* for 15 min, and the supernatant was removed. Finally, 10 µl of Hi-Di Formamide (Applied Biosystems, Foster City, CA, USA) was added to the sediment and heated at 95°C for 2 min. Direct DNA sequencing was performed using a 3730 DNA Analyzer (Applied Biosystems). The electropherogram of the targeted amino acid replacement was analyzed using MEGA 4.0 public domain software (http://www.megasoftware.net/). The unique DNA haplotype sequences were deposited into GenBank (accession number AB634853 - AB634855).

### Geographical analysis

A satellite image of Kenya in Geo-TIFF format (MAP LIBRARY, http://www.maplibrary.org/index.php) was used to map the collection sites. The geographical positions of the collection sites and the allelic frequencies of the L1014S point mutation in larvae were plotted on the map using ArcGIS 9.3 (ESRI Japan Corp, Tokyo, Japan).

## Results

### Distribution of *Anopheles gambiae* s.s. and *An. arabiensis*

Larvae of *An. gambiae* s.l. were collected from 376 breeding sites of which 99 habitats contained *An. gambiae* s.s. (27.3%), 361 contained *An. arabiensis* (96.0%), and 87 contained both species (23.1%), indicating that *An. arabiensis* was broadly distributed in the surveyed region, while the distribution of *An. gambiae* s.s. was sparse.

### Allelic frequency of the L1014S mutation in *Anopheles gambiae* s.s. and *An. arabiensis*

**Figure 1:**
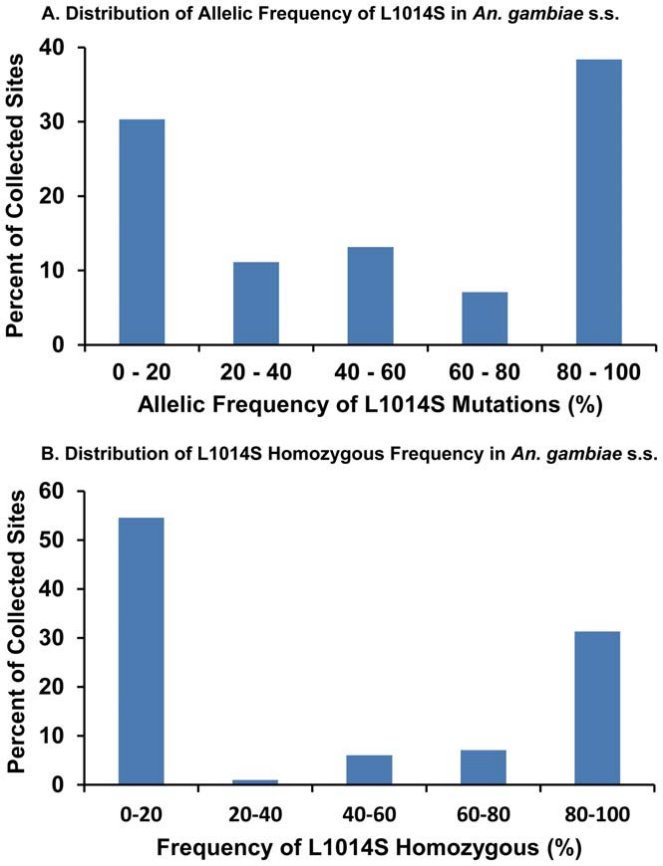
Distribution patterns of the L1014S allelic and homozygous frequencies in *An. gambiae* s.s.

**Figure 2:**
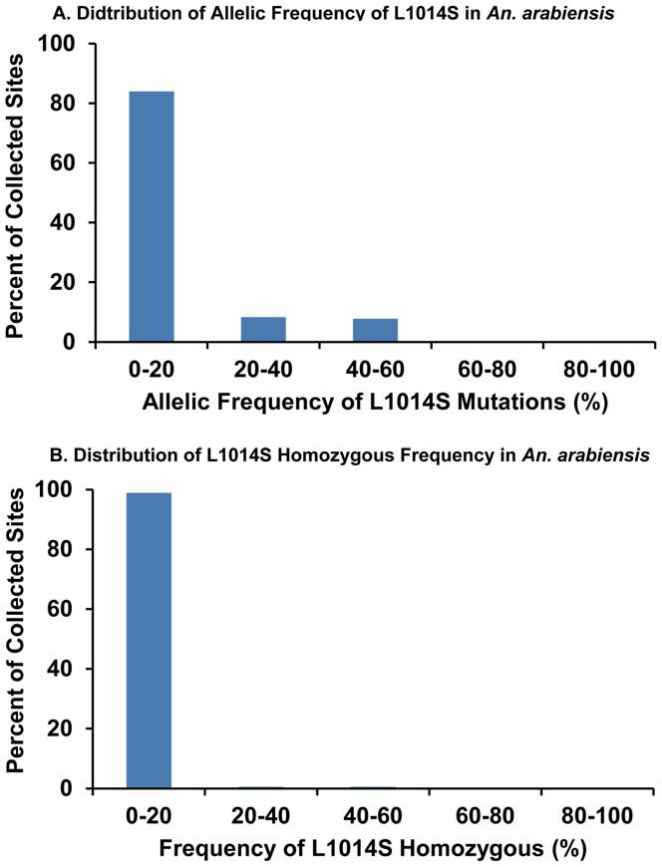
Distribution patterns of the L1014S allelic and homozygous frequencies in *An. arabiensis*.

The allelic and homozygous frequencies of the L1014S mutation in *An. gambiae* s.s. and *An. arabiensis* larvae collected in southern and western regions of Kenya are shown in Figure 1 and 2. The West African mutation L1014F was not detected in our study. Higher allelic and homozygous frequencies of the L1014S mutation were found in *An. gambiae* s.s. than in *An. arabiensis*. Among the 99 sites where *An. gambiae* s.s. larvae were collected, L1014S mutations were found in 72 sites (72.7%), and allelic and homozygous frequencies were >50% at 57 (57.6%) and 44 (44.4%) sites, respectively. At 25 sites (25.3%), all larvae collected were homozygous for the mutation. By contrast, among the 361 sites where *An. arabiensis* larvae were collected, the L1014S mutation was found at 136 sites (37.7%), and allelic and homozygous frequencies were >50% at 22 (6.1%) and 2 (0.55%) sites, respectively. The highest homozygous frequency, 50%, was found at only 2 sites (0.55%). Additionally, the perfectly same intron sequence (length: 57 base) as shown in *An. gambiae* with homozygous *kdr* mutation was found in some *An. arabiensis* samples, indicating the introgression of *kdr* genes between two species.

**Figure 3:**
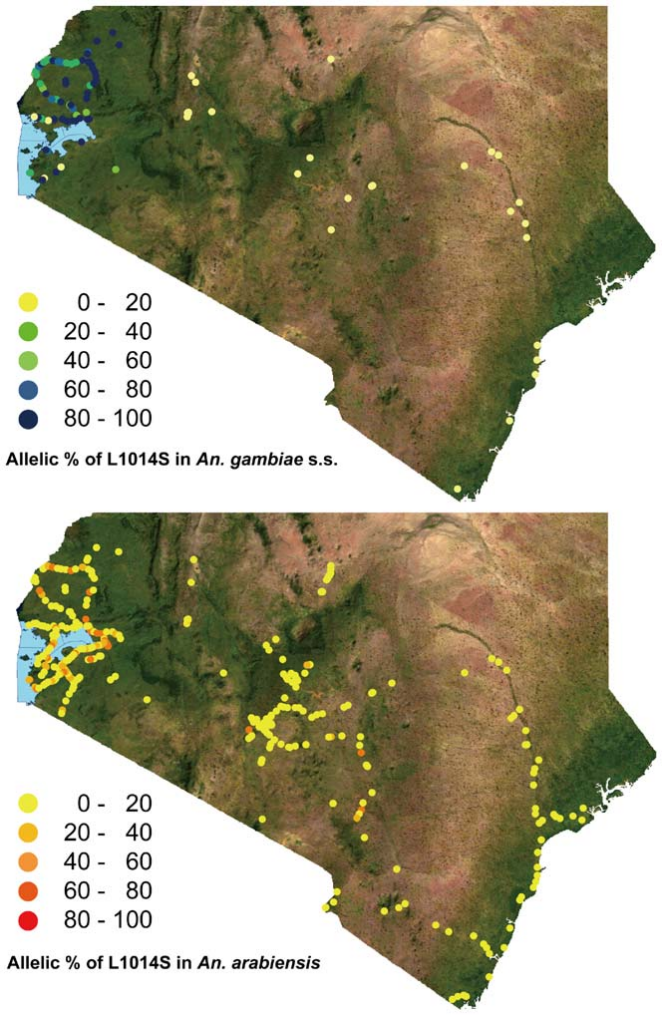
*An. gambiae* s.s. and *An. arabiensis* L1014S allelic frequency distributions in southern and western Kenya.

**Figure 4:**
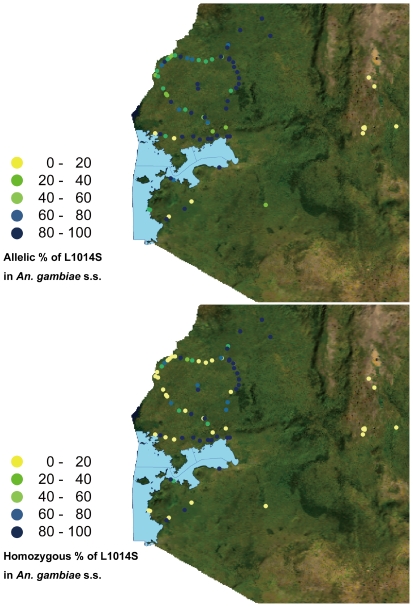
Enlarged images of *An. gambiae* s.s. L1014S allelic and homozygous frequency distributions in western Kenya.

**Figure 5:**
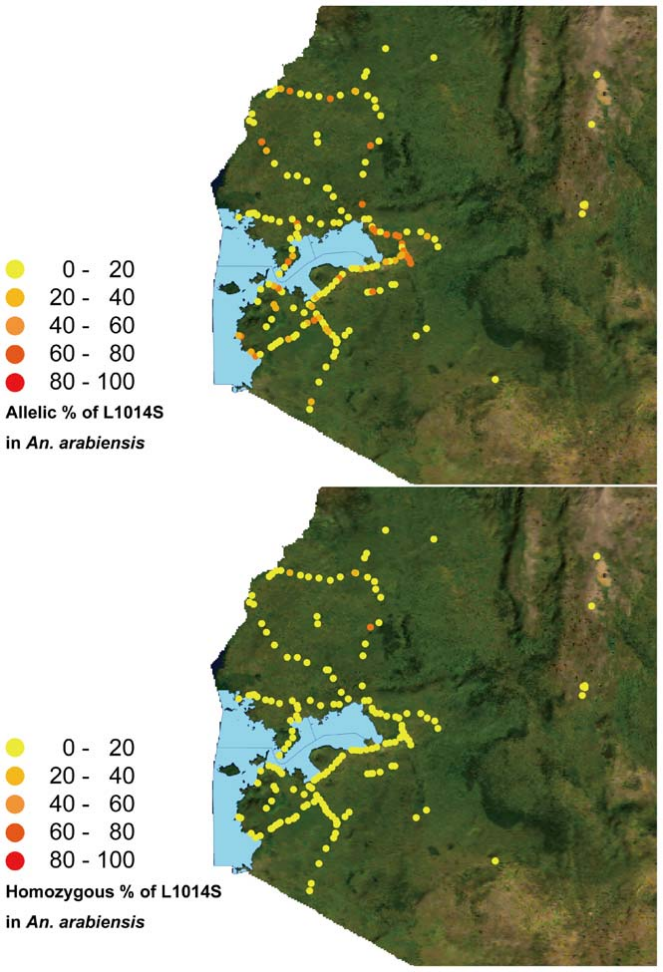
Enlarged images of *An. arabiensis* L1014S allelic and homozygous frequency distributions in western Kenya.

Figure 3 shows the distribution of allelic frequencies of the L1014S mutation in *An. gambiae* s.s. and *An. arabiensis* in southern and western Kenya. The highest allelic frequencies of L1014S in both species seemed to be concentrated in western regions (N 0° 57.416′–S 0° 48.697′, E 34° 9.697′–E 35° 7.986′). Enlarged images of the distributions of allelic and homozygous frequencies of L1014S in the western part of Kenya are shown in Figure 4 and 5. High allelic and homozygous frequencies of L1014S mutations in *An. gambiae* s.s. were found almost uniformly in western highland regions with elevations of >1500 m (Busia, Butere Mumias, Bungoma, Lugari, and Kakamega districts) as well as northern and southern coastal regions of Lake Victoria with elevations of 1100–1300 m (Bondo, Kisumu, Nyando, Rachuonyo, Homa Bay, and Suba districts). Similarly, L1014S mutations in *An. arabiensis* were distributed broadly in the above regions, although the frequencies were lower than those in *An. gambiae* s.s.

## Discussion

In our study, *Anopheles arabiensis* was found to be widely distributed in the southern and western parts of Kenya, while the distribution of *An. gambiae* s.s. was largely restricted to the Western and Nyanza provinces. This distribution pattern seems to be identical to that reported by Okara et al. [20]. Recently, Bayoh et al. [9] reported a historical population decline of *An. gambiae* s.s. associated with increased ITN coverage in western Nyanza province, Kenya. The authors reported that *An. gambiae* s.s. predominated from 1970 to 1988 (approx. 85% of *An. gambiae* s.l. collected indoors) in western Kisumu district. However, the population of *An. gambiae* s.s. decreased relative to that of *An. arabiensis* in 1999, and *An. gambiae* s.l. made up only 1% of indoor collections by 2009. Bayoh et al. [9] also reported the disproportion of the 2 species in western Kenya and found that the frequency of *An. gambiae* s.s. varied by site, with frequencies of <15% at sites west of Kisumu and along the lakeshore (Asembo and Kisian) but >80% at sites further from the lakeshore (Busia, Bungoma, Kakamega, and Malaba). They attributed the decline in *An. gambiae* s.s. at the 2 sites along the lakeshore (Asembo and Kisian) to the rollout of LLITNs. Similar findings in the south of the region on the opposite side of Lake Victoria (Mbita district) were recently reported by Kawada et al. [15]. The present results might indicate that the same decline in the proportion of *An. gambiae* s.s. relative to *An. arabiensis* described above has occurred broadly in southern and western Kenya in the 10 years since the rollout of LLITNs.

It is noteworthy that the L1014S mutation is also unevenly distributed in our study region in both *An. gambiae* s.s. and *An. arabiensis*. The high allelic frequency of L1014S in both species seemed to be concentrated in the western part of the region surveyed, including the highland regions as well as the northern and southern coastal regions of Lake Victoria. These regions have been identified as among Kenya's high vector transmission regions, and more than 50% of the population is exposed to ≥40% *Pf*PR_2–10_ (*Plasmodium falciparum* parasite rate corrected to a standard age range of 2 to less than 10 years old) [21]; therefore, high LLITN or ITN coverage has been implemented [22]. In fact, the percentages of households with at least one LLITN in Nyanza (76.5%) and Western (71.4%) provinces were much higher than those in the other provinces (32.7–66.3%) except for North Eastern province, where coverage is high (73.3%) but we have not performed mosquito sampling (Kenya DHS Final Report, 2009). Moreover, the high population densities of both humans (Gridded population of the world and the global rural-urban mapping project, SEDAC, http://sedac.ciesin.org/gpw/index.jsp) and *An. gambiae* s.s. and *An. arabiensis* mosquitoes [20] in the above regions may have increased the frequency with which vector mosquitoes are exposed to LLITN/ITN, resulting in strong selection pressure by pyrethroids. Mathias et al. [8] reported that the East African *kdr* allele (L1014S) increased in frequency during the past decade among *An. gambiae* s.s. in western Kenya, most of which are now homozygous for the *kdr* allele, concomitant with a regional increase in household ownership of ITNs. The same correlation between the increase in L1014S frequency and increased use of ITN was reported by Stump et al. [7].

This is the first report of the wide distribution of the L1014S mutation in *An. arabiensis*, although the allelic frequencies were lower than in *An. gambiae* s.s. The L1014S and L1014F point mutations have been reported to be rare in *An. arabiensis* [7], [10], [11], [12], [13], [14], [15] with the exception of an Ethiopian case [16], [17], while several cases of P450-related pyrethroid resistance have been reported in this species in Kenya [11], [15]. In prior studies, not a single L1014S allele was found in *An. arabiensis* in Chad [10], Zimbabwe [12], or Malawi [13], only 1 sample heterozygous for L1014S was found in 572 samples in Asembo, Kenya [7] and another in 54 samples in Ahero, Kenya [11], and only 9 samples homozygous (0.04%) and 4 heterozygous (0.02%) for L1014S were reported in 243 *An. arabiensis* in Uganda [14]. The pyrethroid resistance in *An. arabiensis* in the Ethiopian studies [16], [17] is unique as the West African type L1014F mutation was found at a high frequency while the East African type mutation (L1014S) was absent. The authors suggested that the high frequency of such *kdr* mutations could be attributed to the long intensive use of DDT in indoor residual spraying for malaria control and/or to the extensive illegal use of DDT for the control of agricultural pests.

Two hypotheses could be proposed to explain the origin of the *kdr* gene in *An. arabiensis*. The first option is that the same point mutations have developed independently in *An. gambiae* and *An. arabiensis*. The second and most plausible option is that the L1014S point mutation was introgressed from *An. gambiae* by hybridization which has been experimentally demonstrated by previous reports [23], [24], [25]. In fact, the perfectly same intron sequence (length: 57base) as show in *An. gambiae* with homozygous *kdr* mutation was found in some *An. arabiensis* samples in the present study, although there was a diversity in the SNPs and the length of the intron between two species.

Recently, Kawada et al. [15] reported multimodal pyrethroid resistance in 3 major malaria vectors, *An. gambiae* s.s., *An. arabiensis*, and *An. funestus* s.s., in Mbita district, western Kenya. While not a single L1014S allele was detected in the *An. arabiensis* collected (208 females and 77 males), the species had high pyrethroid resistance governed by a P450-related metabolic mechanism. In contrast, significantly higher allelic (>90%) and homozygous (>80%) frequencies of L1014S were detected in *An. gambiae* s.s., most of which were collected in the western islands of Mbita district. The high allelic frequency of the L1014S mutation seemed to be detected equally in *An. gambiae* s.s. collected in the inland regions and the islands, although the numbers collected were small. It is noteworthy that 2 different resistance mechanisms have developed independently in 2 sibling species in the same region. The resistance levels of the *An. arabiensis* collected in the present study are unknown, since no bioassay was performed. The presence of high P450-related pyrethroid resistance in this species in the western region is expected to be comparable to that in Mbita district case [15]. At present, the contribution of the L1014S mutation to resistance in *An. arabiensis* may be negligible, since the homozygous frequency is much lower than in *An. gambiae* s.s. However, the homozygous frequency may increase with continuing selection pressure due to expanded LLITN coverage in the future. Periodic monitoring of both the homozygous frequency of the L1014S mutation and phenotypic pyrethroid resistance in *An. arabiensis* will therefore be essential for the rational and effective control of vector mosquitoes.
